# Asthma, from mild to severe, is an independent prognostic factor for mild to severe Coronavirus disease 2019 (COVID‐19)

**DOI:** 10.1111/crj.13480

**Published:** 2022-02-11

**Authors:** Wang Chun Kwok, Anthony Raymond Tam, James Chung Man Ho, David Chi Leung Lam, Terence Chi Chun Tam, King Pui Florence Chan, Julie Kwan Ling Wang, Mary Sau Man Ip, Ivan Fan Ngai Hung

**Affiliations:** ^1^ Department of Medicine The University of Hong Kong Hong Kong

**Keywords:** asthma, COVID‐19, invasive mechanical ventilation, prognosis

## Abstract

**Background:**

Chronic illnesses were reported to be poor prognostic factors associated with severe illness and mortality in Coronavirus disease 2019 (COVID‐19) infection. The association with asthma, however, is limited and controversial, especially for mild asthma.

**Methods:**

A territory wide retrospective study was conducted to investigate the association between asthma and the prognosis of COVID‐19. All patients with laboratory confirmed in Hong Kong for COVID‐19 from the 23 January to 30 September 2020 were included in the study. Severe diseases were defined as those who develop respiratory complications, systemic complications, and death.

**Results:**

Among the 4498 patients included in the analysis, 165 had asthma, with 141 having mild asthma. Patients with asthma were significantly more likely to require invasive mechanical ventilation (incidence = 17.0% odds ratio [OR] = 4.765, *p* < 0.001), oxygen therapy (incidence = 39.4%, OR = 3.291, *p* < 0.001), intensive care unit admission (incidence = 21.2%, OR = 3.625, *p* < 0.001), and systemic steroid treatment (incidence = 34.5%, OR = 4.178, *p* < 0.001) and develop shock (incidence = 16.4%, OR = 4.061, *p* < 0.001), acute kidney injury (incidence = 6.1%, OR = 3.281, *p* = 0.033), and secondary bacterial infection (incidence = 56.4%, OR = 2.256, *p* < 0.001). They also had significantly longer length of stay. Similar findings were also found in patients with asthma of the Global Initiative for Asthma (GINA) steps 1 and 2 upon subgroup analysis.

**Conclusions:**

Asthma, regardless of severity, is an independent prognostic factor for COVID‐19 and is associated with more severe disease with respiratory and systemic complications.

## Abstract 2

Both mild and severe asthma are independent prognostic factors for Coronavirus disease 2019 (COVID‐19). They are associated with more severe disease with respiratory and systemic complications.

## 1 INTRODUCTION

Coronavirus disease 2019 (COVID‐19) is one of the major healthcare threats in the past decade. Cardiovascular comorbidities and malignancies were reported to be poor prognostic factors associated with severe illness and mortality.^1^, ^2^, ^3^, ^4^, ^5^, ^6^, ^7^

The evidence on asthma and COVID‐19 remains controversial. Some studies did not suggest the association between asthma and risk of hospitalization or worse outcomes among hospitalized patients.^8^, ^9^ Large‐scale study reported that asthma led to poor outcomes of COVID‐19, while underlying asthma, use of asthma medication, and asthma severity were not independent factors for poor clinical outcomes of COVID‐19.^10^ On the other hand, severe asthma, which was defined as defined as asthma with recent use of an oral corticosteroid, was shown to be associated with COVID‐19‐related deaths in another study.^6^ Another cohort study showed that asthma predicted intubation duration^11^ Big data analysis suggested that patients with asthma and COVID‐19 were older and at increased risk due to comorbidity‐related factors.^12^ Systematic reviews and meta‐analyses did not demonstrate the association between asthma and COVID‐19 severity and prognosis.^13^, ^14^

Most of the studies analyzed asthma as a whole and did not separate into subgroups with different severity. The definition of severe asthma in Williamson et al. is also not based on the Global Initiative for Asthma (GINA) guideline^15^ or by the European Respiratory Society/American Thoracic Society guideline.^16^ The impact from mild asthma, which contributes to the majority of asthma cases, is also not assessed in previous studies. The association of asthma and prognosis from previous studies could be mediated by the severe asthma cases within the cohorts, with more severe asthma cases in the cohorts may bias the result to be positive and vice versa.

In this current study, we investigated the association between asthma of different severity based on GINA definition and the prognosis of COVID‐19, from a territory wide cohort.

## 2 MATERIALS AND METHODS

A territory wide retrospective study was conducted to investigate the association between asthma and the prognosis of COVID‐19. Patients hospitalized for COVID‐19 in public hospitals in Hong Kong for COVID‐19 from 23 January to 30 September 2020 were included. According to public health policy in Hong Kong, all patients with COVID‐19 regardless of disease severity need to be hospitalized in public hospital or community treatment facility which are managed by the Hospital Authority under isolation order from the government. Patients with moderate to severe disease are managed in the isolation ward of acute hospital, while mild and asymptomatic cases are managed in Hong Kong Infection Control Centre. Home‐based management is not adopted in Hong Kong. Patients are only allowed to be discharged from hospital when repeated respiratory specimens were negative for SAR‐CoV2 and the isolation order is removed. Patients were identified from the Clinical Data Analysis and Reporting System (CDARS) of the Hospital Authority. Cases with COVID‐19 were identified by the International Classification of Diseases, Ninth Revision code of 519.8. CDARS is an electronic healthcare database managed by the Hospital Authority which covers 90% of healthcare services of Hong Kong, as well as managing all patients with COVID‐19 in Hong Kong. The study was approved by the Institutional Review Board of the University of Hong Kong and the Hospital Authority Hong Kong West Cluster (UW 21‐055).

Inclusion criteria include age of 18 years old or above with laboratory reverse transcription polymerase chain reaction (RT‐PCR) confirmed COVID‐19. Exclusion criteria include patients with chronic obstructive pulmonary disease and bronchiectasis.

The primary outcome is respiratory failure that requires invasive mechanical ventilation. The secondary outcomes include need of oxygen therapy for respiratory failure, require systemic steroid treatment for COVID‐19, intensive care unit admission, development of acute respiratory distress syndrom (ARDS), shock, development of acute kidney injury, in‐hospital mortality, 30‐day mortality, and secondary bacterial and viral infections.

Mild asthma is defined as patients that have asthma controlled on GINA steps 1 and 2 therapy. Moderate to severe asthma was defined as asthma controlled on GINA steps 3 to 5 therapy.^17^

### 2.1 Statistical analysis

The demographic and clinical data were described in actual frequency or mean ± SD. Baseline demographic and clinical data were compared between the patients with or without asthma by independent *t‐*test. To identify whether asthma is associated with severe disease, univariate logistic regression analyses were performed. Multiple logistic regression modeling was used to take into account of potential confounders including age, comorbidities, which are known poor prognostic factors of COVID‐19, as well as the treatment patient received for treatment of COVID‐19. Length of stay between the two groups was compared by log‐linear regression. The statistical significance was determined at the level of *p* = 0.05. All the statistical analyses will be done using the 26th version of SPSS statistical package.

## 3 RESULTS

From 23 January to 30 September 2020, a total of 4548 adult patients with COVID‐19 were hospitalized for COVID‐19 in public hospitals in Hong Kong. Forty patients with COPD and 10 patients with bronchiectasis were excluded. Among the 4498 patients included in the analysis, there were 2195 (48.8%) male patients, with mean age of 47.0. There were 165 patients with asthma in the cohort. Among the patients with asthma, 120 (72.7%) were on GINA step 1 treatment, 21 (12.7%) were on GINA step 2 treatment, 6 (3.6%) were on GINA step 3 treatment, 16 (9.7%) were on GINA step 4 treatment, and 2 (1.2%) on GINA step 5 treatment. The baseline demographics of the patients were listed in Tables 1 and 2 and supporting information Table S1.

**TABLE 1:** Baseline demographic of included patients

	Patients without asthma (*n* = 4333)	Patients with asthma (*n* = 165)	*p* value
Age, mean ± SD (range)	46.5 ± 17.8 (18–102)	57.8 ± 19.5 (19–68)	<0.001
Sex			0.003
Male	2096 (48.4%)	99 (60.0%)	
Female	2237 (51.6%)	66 (40.0%)	
Hypertension	876 (20.2%)	68 (41.2%)	<0.001
Diabetes mellitus	476 (11.0%)	38 (23.0%)	<0.001
Hyperlipidemia	536 (12.4%)	48 (29.1%)	<0.001
Ischemic heart disease	260 (6.0%)	25 (12.5%)	<0.001
Atrial fibrillation	70 (1.6%)	16 (9.7%)	<0.001
Gout	82 (1.9%)	28 (4.8%)	0.017
Malignancies	34 (0.8%)	6 (3.6%)	0.000
eGFR, mean ± SD (range)	84.5 ± 12.6 (3–90)	78.3 ± 17.0 (27–90)	0.000

**TABLE 2:** Baseline demographic of patients with mild and moderate to severe asthma

	Patients with mild asthma (*n* = 141)	Patients with moderate to severe asthma (*n* = 24)	*p* value
Age, mean ± SD (range)	57.1 ± 18.6 (20–98)	54.3 ± 19.4 (19–93)	0.506
Sex			0.134
Male	80 (56.7%)	18 (75.0%)	
Female	61 (43.3%)	6 (25.0%)	
Hypertension	54 (38.3%)	10 (41.7%)	0.857
Diabetes mellitus	33 (23.4%)	4 (16.7%)	0.416
Hyperlipidemia	38 (27.0%)	10 (41.7%)	0.176
Ischemic heart disease	20 (14.2%)	2 (8.3%)	0.403
Atrial fibrillation	11 (7.8%)	2 (8.3%)	0.968
Gout	7 (5.0%)	1 (4.2%)	0.839
Malignancies	6 (4.3%)	0 (0%)	0.294
eGFR, mean ± SD (range)	79.7 ± 16.3 (27–90)	80.2 ± 17.7 (46–116)	0.921

### 3.1 Disease severity and complications for patients with or without asthma

On univariate logistic regression, patients with asthma were significantly more likely to have severe disease and develop complications, including invasive mechanical ventilation, oxygen therapy, and intensive care unit admission with odds ratios (OR) of 7.774 (95% CI = 4.966–12.168, *p*‐value < 0.001), 4.735 (95% CI = 3.420–6.556, *p*‐value < 0.001), and 6.977 (95% CI = 4.653–10.461, *p*‐value < 0.001), respectively. Patients with asthma were also significantly more likely to require systemic corticosteroid treatment, develop ARDS, shock, acute kidney injury (AKI), secondary bacterial infection, in‐patient mortality, and 30‐day mortality. The results are summarized in the supporting information Tables S2 to S4.

Multivariate logistic regression was done to adjust for reported poor prognostic factor including age, hypertension, diabetes mellitus, hyperlipidemia, ischemic heart disease, atrial fibrillation, malignancies, baseline estimated glomerular filtration rate (EGFR) as well as the treatment patients received for COVID‐19, in which some of the factors adjusted in multivariate logistic regression were also noted to have difference among the two groups. Patients with asthma were significantly more likely to require invasive mechanical ventilation with OR of 4.765 (95% CI = 2.438–9.311, *p*‐value < 0.001), oxygen therapy with OR of 3.291 (95% CI = 2.032–5.330, *p*‐value < 0.001), and intensive care unit admission with OR of 4.086 (95% CI = 2.245–7.437, *p*‐value < 0.001.) Patients with asthma were also significantly more likely to systemic corticosteroid treatment, develop shock, develop acute kidney injury, and develop secondary bacterial infection, as shown in the supporting information Tables S3 and S4.

The length of stay was significantly longer among patients with asthma than without asthma. The mean length of stay was 19.1 ± 16.2 for patients with asthma and 13.0 ± 10.8 days for patients without asthma, with *p* < 0.001.

### 3.2 Disease severity and complications for patients with mild asthma and without asthma

Mild asthma accounts for the majority of asthma cases in our cohort. Univariate logistic regression was used to compare the disease severity and complications for patients with mild asthma and without asthma. Patients with mild asthma were significantly more likely to require invasive mechanical ventilation, oxygen therapy, and intensive care unit admission with OR of 6.581 (95% CI = 3.961–10.933, *p*‐value < 0.001), 7.236 (95% CI = 4.955 10.481, *p*‐value < 0.001), and 6.713 (95% CI = 4.247–10.609, *p*‐value < 0.001), respectively. Patients with mild asthma were significantly more likely to require systemic corticosteroid treatment, develop ARDS, develop shock, develop AKI, develop secondary bacterial infection, have in‐patient mortality, and 30‐day mortality. The results are summarized in Tables 3 and 4.

**TABLE 3:** Odds ratios of respiratory complications from COVID‐19 for with mild asthma and without asthma

Complications	Univariate analysis, odds ratios, and 95% CI	*p* value	Multivariate analysis^a^, odds ratios, and 95% CI	*p* value
Invasive mechanical ventilation^b^	6.581 (3.961–10.933)	<0.001	4.660 (2.293–9.472)	<0.001
Invasive mechanical ventilation >95 h	0	0.996		
Oxygen therapy for respiratory failure^b^	7.236 (4.955 10.481)	<0.001	3.665 (2.187–6.142)	<0.001
Require systemic steroid treatment^b^	4.634 (3.250–6.609)	<0.001	3.155 (1.778–5.598)	<0.001
ARDS	4.141 (1.738–9.869)	0.001	2.002 (0.733–5.471)	0.176

^a^ Adjustment done for confounders including age, hypertension, diabetes mellitus, hyperlipidemia, ischemic heart disease, gout, atrial fibrillation, malignancies, EGFR, and the treatment for COVID‐19.

^b^ Factors that are statistically significant after adjustment for confounders.

**TABLE 4:** Odds ratios of systemic complications from COVID‐19 for mild asthma and without asthma

Complications	Univariate analysis, odds ratios, and 95% CI	*p* value	Multivariate analysis^a^, odds ratios, and 95% CI	*p* value
Intensive care unit admission^b^	6.713 (4.247–10.609)	<0.001	4.215 (2.244–7.916)	<0.001
Shock^b^	6.381 (3.814–10.678)	<0.001	4.031 (2.206–8.023)	<0.001
Acute kidney injury^b^	11.158 (4.732–26.309)	<0.001	3.654 (1.205–11.079)	0.022
Secondary bacterial infection^b^	2.809 (1.980–3.985)	<0.001	2.468 (1.506–4.045)	<0.001
Secondary viral infection	1.198 (0.424–3.392)	0.733		
In‐patient mortality	6.624 (2.918–15.026)	<0.001	1.446 (0.445–4.702)	0.540
30‐day mortality	9.194 (3.859–21.904)	<0.001	2.122 (0.634–7.106)	0.222

^a^ Adjustment done for confounders including age, hypertension, diabetes mellitus, hyperlipidemia, ischemic heart disease, gout, atrial fibrillation, malignancies, EGFR and the treatment for COVID‐19.

^b^ Factors that are statistically significant after adjustment for confounders.

Multivariate logistic regression was done to adjust for reported poor prognostic factor, baseline EGFR, and the treatment patients received for COVID‐19. Patients with mild asthma were significantly more likely to require invasive mechanical ventilation with OR of 4.660 (95% CI = 2.293–9.472, *p*‐value < 0.001), oxygen therapy with OR of 3.665 (95% CI 2.187–6.142, *p‐*value < 0.001), and intensive care unit admission with OR of 4.215 (2.244–7.916, *p*‐value < 0.001). Patients with mild asthma were also significantly more likely to require systemic corticosteroid treatment, develop shock, develop AKI, and develop secondary bacterial infection, as shown in Tables 3 and 4.

The length of stay was significantly longer among patients with mild asthma than those without asthma. The mean length of stay was 18.7 ± 16.3 for patients with mild asthma and 13.0 ± 10.8 days for patients without asthma, with *p* < 0.001.

As systemic corticosteroid has the role in controlling asthmatic attack while early use of high dose of systemic corticosteroid may pose higher risk of complications from COVID‐19 infection, analysis on the association of timing of systemic corticosteroid is assessed. The median time to initiation of systemic corticosteroid was 6 (interquartile range [IQR] = 7) days and 6 (IQR = 6) days for patients without and with asthma. The timing of initiation of systemic corticosteroid was not found to be associated with disease severity and complications including invasive mechanical ventilation with OR of 1.007 (95% CI = 0.980–1.034, *p* = 0.607), oxygen therapy (OR = 0.966, 95% CI = 0.892–1.046, *p* = 0.396), intensive care unit admission (OR = 1.014, 95% CI = 0.990–1.038, *p* = 0.265), develop shock (OR = 0.997, 95% CI = 0.968–1.026, *p* = 0.817), develop AKI (OR = 1.011, 95% CI = 0.971–1.052, *p* = 0.596), develop secondary bacterial infection (OR = 1.024, 95% CI = 0.983–1.067, *p* = 0.258), have in‐patient mortality (OR = 0.986, 95% CI = 0.945–1.029, *p* = 0.510), and 30‐day mortality (OR = 0.960, 95% CI = 0.905–1.019, *p* = 0.180).

### 3.3 Disease severity and complications for patients with moderate to severe asthma and without asthma

On univariate logistic regression, patients with moderate to severe asthma were significantly more likely to require invasive mechanical ventilation with OR of 5.409 (95% CI = 1.590–18.401, *p‐*value 0.007), intensive care unit admission with OR of 3.685 (95% CI = 1.088–12.481, *p*‐value 0.036) and develop shock with OR of 5.080 (95% CI = 1.495–17.266, *p*‐value = 0.009). On multivariate logistic regression was done to adjust for reported poor prognostic factor, baseline EGFR, and the treatment patients received for COVID‐19; patients with moderate to severe asthma were significantly more likely to require invasive mechanical ventilation with OR of 5.857 (95% CI = 1.017–33.742, *p*‐value = 0.048).

The length of stay was significantly longer among patients with moderate to severe asthma than those without asthma. The mean length of stay was 21.7 ± 15.6 for patients with moderate to severe asthma and 13.0 ± 10.8 days for patients without asthma, with *p* < 0.001.

The results are summarized in Tables 5 and 6.

**TABLE 5:** Odds ratios of respiratory complications from COVID‐19 for patients with moderate to severe asthma and without asthma

Complications	Univariate analysis, odds ratios, and 95% CI	*p* value	Multivariate analysis^a^, odds ratios, and 95% CI	*p* value
Invasive mechanical ventilation^b^	5.409 (1.590–18.401)	0.007	5.857 (1.017–33.742)	0.048
Invasive mechanical ventilation >95 h	3.301 (0.403–22.806)	0.281		
Oxygen therapy for respiratory failure	1.920 (0.714–5.164)	0.196		
Require systemic steroid treatment	2.087 (0.776–5.615)	0.145		
ARDS	2.675 (0.356–20.087)	0.339		

^a^ Adjustment done for confounders including age, hypertension, diabetes mellitus, hyperlipidemia, ischemic heart disease, gout, atrial fibrillation, malignancies, EGFR and the treatment for COVID‐19.

^b^ Factors that are statistically significant after adjustment for confounders.

**TABLE 6:** Odds ratios of systemic complications from COVID‐19 for patients with moderate to severe asthma and without asthma

Complications	Univariate analysis, odds ratios, and 95% CI	*p* value	Multivariate analysis^a^, odds ratios, and 95% CI	*p* value
Intensive care unit admission	3.685 (1.088–12.481)	0.036	3.455 (0.622–19.211)	0.157
Shock	5.080 (1.495–17.266)	0.009	4.850 (0.886–26.551)	0.069
Acute kidney injury	0	0.998		
Secondary bacterial infection	1.665 (0.738–3.759)	0.220		
Secondary viral infection	0	0.998		
In‐patient mortality	3.190 (0.424–24.016)	0.260		
30‐day mortality	4.125 (0.545–31.203)	0.170		

^a^ Adjustment done for confounders including age, hypertension, diabetes mellitus, hyperlipidemia, ischemic heart disease, gout, atrial fibrillation, malignancies, EGFR and the treatment for COVID‐19.

## 4 DISCUSSION

Our study suggested that both mild and moderate to severe asthma are associated with severe COVID‐19 disease. Patients with mild and moderate to severe asthma had increased risks of severe respiratory failure that required invasive mechanical ventilation, while patients with mild asthma had increased risks in developing other respiratory and systemic complications as well. Patients with both mild and moderate to severe asthma also had significantly loner length of stay. While cardiovascular comorbidities and malignancies were reported to be associated with severe disease, these factors may represent older patients with worse general health and some degree of immunosuppression. For asthma, the results from previous studies were controversial and there has not been a universally accepted definition of asthma severity in these studies. The difference for asthma and other chronic respiratory disease is that it ranges from mild cases that only need as need reliever, to severe cases which need long‐term systemic steroid or biologics, and the disease can be improved with adequate and appropriate treatment. Mild asthma contributes to the majority of patients with asthma. Our studies suggest that patients with both mild and moderate to severe asthma are associated with severe disease. Asthma patients also had longer length of stay.

While previous studies have not examined on this association, our study is more comprehensive in including all patients with confirmed COVID‐19 in Hong Kong within the captioned period as all patients with COVID‐19 regardless of disease severity need to be hospitalized under isolation order from the government. This arrangement is rarely adopted in other countries. Such arrangement allows our cohort to include patients ranging from mild to fatal infection with all comorbidities, investigation results, complications, and treatment details available, which is the strength of our study. This allows comprehensive examination of the association of asthma of different severity and severity of COVID‐19, in contrast to studies conducted in other countries in which patients with moderate to severe infections are hospitalized.^17^ Using a universally accepted definition of mild and severe asthma is another strength of our study. For mortality, the overall mortality is low in the captioned period in Hong Kong. There were only 70 reported deaths among this cohort, with mortality rate of 1.56%. While there is a trend to suggest that asthma cases had a higher mortality, it did not reach satirical significance, likely related to the low number of events within the cohort. Another reason to suggest this is that mortality from COVID‐19 is more likely to be related to other comorbidities than asthma. This is supported by the finding in our study that the associations between mild asthma and in‐patient mortality/30‐day mortality were significant at univariate logistic regression but not multivariate logistic regression. Moreover, patients with asthma, regardless of the severity, upon development of severe disease and complications, as long as not having concomitant comorbidity, may still be able to survive with severe COVID‐19 infection. This is likely because asthma, when compared with other cardiovascular comorbidities and malignancies, is not an immunocompromised state.

The results of our study have important implication in the management of patients with asthma and COVID‐19. As vaccines are now available,^18^, ^19^, ^20^, ^21^, ^22^, ^23^, ^24^ patients with asthma are strongly indicated to receive COVID‐19 vaccines regardless of the severity of asthma if not contraindicated. Receiving COVID‐19 vaccine will have dual benefits for patients with asthma, in preventing COVID‐19 infection which is shown to have various complications from our study, as well asthma exacerbation, which is commonly triggered by viral respiratory tract infections.^25^, ^26^, ^27^ The benefits of COVID‐19 vaccine are more pronounced among patients with severe asthma who require repeated courses of systemic corticosteroid for asthmatic exacerbation and maintenance steroid. Patients who are on immunosuppressant including steroid have an increased risk of COVID‐19 and its complications.^28^, ^29^ Preventing the development of COVID‐19, especially severe infection, will be crucial for patients with severe asthma dependent on long‐term steroid. Patients with mild asthma should also be considered to have in‐patient management as they are prone to develop severe COVID‐19 infection, instead of out‐patient management.^8^ They should also be considered for early treatment of COVID‐19 for the same reason.

The limitation in our study includes lack of data of viral load for the patients. But the need for treatment for COVID‐19 was potential surrogates, as asymptomatic patients with low viral load would not need treatment. To better assess the association reported in our study, which is a post hoc comparison, conducting a prospective study analysis would be worthwhile. While GINA steps were used in our study to categorize the severity of asthma, which is one of the most commonly used definitions, this only reflects the level of control of asthma by the medication. A more sophisticated study that includes biological and physiological measures to categorize the severity, such as spirometry data and fractional exhaled nitric oxide (FeNO), would be able to provide more information on this.

## 5 CONCLUSION

Asthma, regardless of severity, is an independent prognostic factor for COVID‐19 and is associated with more severe disease with respiratory and systemic complications.

## GUARANTOR OF THE ARTICLE

Prof. Ivan Fan Ngai Hung.

## AKNOWLEDGMENT

We acknowledge the medical and nursing staff in the Hospital Authority of Hong Kong in managing patients with COVID‐19 infected patients.

## CONFLICT OF INTEREST

The authors declares that there is no conflict of interest.

## FUNDING INFORMATION

Nil.

## ETHICS STATEMENT

The study is performed in accordance to the Declaration of Helsinki.

## AUTHOR CONTRIBUTIONS

Dr. Wang Chun Kwok was involved with study concept and design, analysis and interpretation of data, acquisition of data, drafting of manuscript, and approval of the final version of the manuscript. Dr. Anthony Raymond Tam, King Pui Florence Chan, Terence Chi Chun Tam Chun Leung, James Chung Man Ho, David Chi Leung Lam, Julie Kwan Ling Wang, and Prof. Mary Sau Man Ip were involved with critical revision of the manuscript for important intellectual content and approval of the final version of the manuscript. Prof. Ivan Fan Ngai Hung was involved with the study concept and design, analysis and interpretation of data, drafting of manuscript, critical revision of the manuscript for important intellectual content, study supervision, and approval of the final version of the manuscript.

## Supporting information

Supplementary Table S1: COVID‐19 treatment regimens of the included patients
Supplementary Table S2: Incidence of respiratory and systemic complications of the included patients
Supplementary Table S3: Odds ratios of respiratory complications from COVID‐19 for patients with or without asthma
Supplementary Table S4: Odds ratios of systemic complications from COVID‐19 for patients with or without asthma

## Data Availability

Research data are not shared.
