# Supplementary material for: Asthma, from mild to severe, is an independent prognostic factor for mild to severe Coronavirus disease 2019 (COVID‐19)
Source: Clin Respir J. 2022 Feb 11;16(4):293–300. doi: 10.1111/crj.13480 (PMC9060123; doi:10.1111/crj.13480)
Supplement: Supplementary file 1 — Supplementary Table S1: COVID‐19 treatment regimens of the included patients Supplementary Table S2: Incidence of respiratory and systemic complications of the included patients Supplementary Table S3: Odds ratios of respiratory complications from COVID‐19 for patients with or without asthma Supplementary Table S4: Odds ratios of systemic complications from COVID‐19 for patients with or without asthma [file CRJ-16-293-s001.docx]

Supplementary table 1: COVID-19 treatment regimens of the included patients

|  | Patients without asthma  (n = 4333) | Patients with asthma  (n = 165) | p value |
| --- | --- | --- | --- |
| No. | 2184 (50.4%) | 56 (33.9%) | 0.962 |
| Interferon beta-1b, lopinavir–ritonavir, and ribavirin | 440 (10.2%) | 35 (21.2%) | < 0.001 |
| Lopinavir–ritonavir and ribavirin | 206 (4.8%) | 9 (5.5%) | 0.305 |
| Interferon beta-1b and ribavirin | 429 (9.9%) | 19 (11.5%) | 0.128 |
| Interferon beta-1b and lopinavir–ritonavir | 786 (18.1%) | 28 (17.0%) | 0.287 |
| Ribavirin | 26 (0.6%) | 0 (0%) | 0.715 |
| Interferon beta-1b | 70 (1.6%) | 7 (4.2%) | < 0.001 |
| Lopinavir–ritonavir | 58 (1.3%) | 1 (1.4%) | 0.858 |
| Interferon beta-1b and clofazamine | 70 (1.6%) | 7 (4.2%) | 0.715 |
| Clofazamine | 9 (0.2%) | 0 (0%) | 0.891 |
| Interferon beta-1b and hydroxychloroquine | 52 (1.2%) | 3 (1.8%) | 0.205 |
| Azithromycin and hydroxychloroquine | 3 (0.1%) | 0 (0%) | 0.962 |

Supplementary table 2: Incidence of respiratory and systemic complications of the included patients

|  | No asthma (n = 4333) | Asthma (n = 165) | p value |
| --- | --- | --- | --- |
| Respiratory failure requiring oxygen therapy | 523 (12.1%) | 65 (39.4%) | < 0.001 |
| Invasive mechanical ventilation | 111 (2.6%) | 28 (17.0%) | < 0.001 |
| Invasive mechanical ventilation of more than 95 hours | 61 (1.4%) | 1 (0.6%) | 0.386 |
| Extra-corporeal membranous oxygenation | 3 (0.1%) | 0 (0%) | 0.735 |
| Require systemic steroid treatment | 486 (11.2%) | 57 (34.5%) | < 0.001 |
| Intensive care unit admission | 161 (3.7%) | 35 (21.2%) | < 0.001 |
| ARDS | 69 (1.6%) | 8 (4.8%) | 0.002 |
| Shock | 118 (2.7%) | 27 (16.4%) | < 0.001 |
| Acute kidney injury | 34 (0.8%) | 10 (6.1%) | < 0.001 |
| Secondary bacterial infection | 1303 (30.1%) | 93 (56.4%) | < 0.001 |
| Secondary viral infection | 79 (1.8%) | 4 (2.4%) | 0.573 |
| In-patient mortality | 60 (1.4%) | 10 (6.1%) | < 0.001 |
| 30 day mortality | 46 (1.1%) | 10 (6.1%) | < 0.001 |
| Mean length of stay +/- SD (Days) | 13.0 +/- 10.8 | 19.1 +/- 16.2 | < 0.001 |

Supplementary table 3: Odds ratios of respiratory complications from COVID-19 for patients with or without asthma

| **Complications** | **Univariate analysis**  **Odds ratios and 95% CI** | **p value** | **Multivariate analysis**#  **Odds ratios and 95% CI** | **p value** |
| --- | --- | --- | --- | --- |
| Invasive mechanical ventilation * | 7.774 (4.966 – 12.168) | < 0.001 | 4.765 (2.438 – 9.311) | < 0.001 |
| Invasive mechanical ventilation > 95 hours | 0.427 (0.059 – 3.099) | 0.400 |  |  |
| Oxygen therapy for respiratory failure* | 4.735 (3.420 – 6.556) | < 0.001 | 3.291 (2.032 – 5.330) | < 0.001 |
| Require systemic steroid treatment* | 4.178 (2.990 – 5.837) | < 0.001 | 2.945 (1.723 – 5.035) | < 0.001 |
| ARDS | 3.149 (1.489 – 6.661) | 0.003 | 2.188 (0.859 – 5.575) | 0.101 |

*Factors that are statistically significant after adjustment for confounders

#Adjustment done for confounders including age, hypertension, diabetes mellitus, hyperlipidemia, ischemic heart disease, gout, atrial fibrillation, malignancies, EGFR and the treatment for COVID-19

Supplementary table 4: Odds ratios of systemic complications from COVID-19 for patients with or without asthma

| Complications and length of stay | Univariate analysis  Odds ratios and 95% CI | p value | Multivariate analysis | p value |
| --- | --- | --- | --- | --- |
| Intensive care unit admission* | 6.977 (4.653 – 10.461) | < 0.001 | 4.086 (2.245 – 7.437) | < 0.001 |
| Shock* | 6.989 (3.049 – 11.171) | < 0.001 | 4.061 (2.117 – 7.787) | < 0.001 |
| Acute kidney injury* | 8.157 (3.959 – 16.810) | < 0.001 | 3.281 (1.099 – 9.796) | 0.033 |
| Secondary bacterial infection* | 3.004 (2.193 – 4.113) | < 0.001 | 2.256 (1.431-3.557) | <0.001 |
| Secondary viral infection | 1.338 (0.484 – 3.698) | 0.575 |  |  |
| In-patient mortality | 3.180 (0.711 – 14.231) | 0.130 |  |  |
| 30 day mortality | 6.013 (2.979 – 12.137) | < 0.001 | 2.578 (0.839 – 7.920) | 0.098 |

*Factors that are statistically significant after adjustment for confounders

#Adjustment done for confounders including age, hypertension, diabetes mellitus, hyperlipidemia, ischemic heart disease, gout, atrial fibrillation, malignancies, EGFR and the treatment for COVID-19
